# Reply to Dr. Page

**Published:** 1893-09

**Authors:** Charles H. Taft

**Affiliations:** Chicago


### Domestic Correspondence.

### REPLY TO DR. PAGE.

CHICAGO, August 15, 1893.

EDWARD PAGE, D.M.D., CHARLESTOWN, MASS.:

DEAR DOCTOR,—I have read with much interest your recent paper on "Amalgam as a Filling-Material," published in the August issue of the INTERNATIONAL DENTAL JOURNAL, and while I am willing to believe you are entirely honest and sincere in your opinion that amalgam fillings prove no obstacle very often to the physician in the cure of the sick, I cannot give you credit for any sincerity in what you say is "your desire to get at the truth of this matter," and I doubt very much if you have ever removed in one single instance the amalgam fillings from the teeth of any patient that may have been sent to you with such instructions from the physician simply to satisfy yourself whether or not there was any subsequent improvement in the patient's condition.

You say you are "a student seeking for truth in every department of life, and desire to approach this subject of amalgam in an *impartial* spirit, without fear or prejudice." This being the case, would it not be well, when you have the opportunity freely given you, to at least be willing to meet a man in friendly debate who could present the subject in a different light from that in which you see it, and to hear what he has to say on the subject? Would it not be well, furthermore, instead of making simple assertions and denials, which prove nothing, to take up and to investigate for yourself in a practical and scientific manner, and without prejudice, whether the statement which physicians make upon the point at issue is correct or not?

One who was cognizant of the recent action taken by the officers and executive committee of the Massachusetts Dental Society, in which I am informed you took an important part in refusing a hearing to my paper, would gather from your paper that you were more than desirous to hear the reasons why physicians advise the removal of amalgams in many instances before they can effect a cure, for you say, "Now, if there is any serious or valid reason why we should not use it; I would be glad to know it, not believe it, but have scientific evidence given supported by facts so abundant that there would be no reasonable doubt about it." Allow me to say that a presentation of those reasons and facts were, as you well know, ready to be given you at the recent meeting of the Massachusetts Society had you really desired to hear them.

Again you say, "Therefore let those few persons who object to it on any ground give us and the *world* some good plain scientific reasons to prove it unfit to use in our practice. If there is any one who can show cause why amalgam should not be used as a filling-material, let us calmly listen to substantial proof and scientific demonstration."

Now, doctor, allow me to suggest that, considering the facts were all ready and waiting for a hearing, and which, partly by your own vote and influence, as is well known, were refused a hearing, such sentiments as the ones I have quoted from your paper are neither manly nor sincere.

Fair play is a thing that all honest men admire, and a manly and friendly interchange of opinion upon subjects of common interest among men who differ should always be encouraged in any society that claims to be scientific, and to any man in joining a society and bringing before it anything of interest can be attributed, in all probability, no baser motive than one of friendly interest and helpfulness towards his brother practitioners.

Before a member in good standing is again invited to read a paper before the Massachusetts Dental Society and refused a hearing because he insists upon the natural right and privilege of every man to say for himself, without assistance from others, what shall be the title of any subject upon which it is always his prerogative to write, it is to be hoped the Society will have learned a lesson in the rules which govern fair play.

Very truly yours,

CHARLES H. TAFT.

